# Biased data, biased AI: deep networks predict the acquisition site of TCGA images

**DOI:** 10.1186/s13000-023-01355-3

**Published:** 2023-05-17

**Authors:** Taher Dehkharghanian, Azam Asilian Bidgoli, Abtin Riasatian, Pooria Mazaheri, Clinton J. V. Campbell, Liron Pantanowitz, H. R. Tizhoosh, Shahryar Rahnamayan

**Affiliations:** 1 University Health Network, Toronto, ON Canada; 2 Department of Pathology and Molecular Medicine, Faculty of Health Science, McMaster University, Hamilton, ON Canada; 3 Nature Inspired Computational Intelligence (NICI), Ontario Tech University, Oshawa, ON Canada; 4 Nature Inspired Computational Intelligence (NICI) Lab, Department of Engineering, Brock University, 1812 Sir Isaac Brock Way, St. Catharines, ON L2S 3A1 Canada; 5 Bharti School of Engineering and Computer Science, Laurentian University, Sudbury, ON Canada; 6 KIMIA Lab, University of Waterloo, Waterloo, ON Canada; 7 William Osler Health System, Brampton, ON Canada; 8 Department of Pathology, University of Michigan, Ann Arbor, MI USA; 9 Rhazes Lab, Department of Artificial Intelligence and Informatics, Mayo Clinic, Rochester, MN USA

**Keywords:** Digital pathology, Deep Learning, AI bias, AI ethics, TCGA, Cancer

## Abstract

**Background:**

Deep learning models applied to healthcare applications including digital pathology have been increasing their scope and importance in recent years. Many of these models have been trained on The Cancer Genome Atlas (TCGA) atlas of digital images, or use it as a validation source. One crucial factor that seems to have been widely ignored is the internal bias that originates from the institutions that contributed WSIs to the TCGA dataset, and its effects on models trained on this dataset.

**Methods:**

8,579 paraffin-embedded, hematoxylin and eosin stained, digital slides were selected from the TCGA dataset. More than 140 medical institutions (acquisition sites) contributed to this dataset. Two deep neural networks (DenseNet121 and KimiaNet were used to extract deep features at 20× magnification. DenseNet was pre-trained on non-medical objects. KimiaNet has the same structure but trained for cancer type classification on TCGA images. The extracted deep features were later used to detect each slide’s acquisition site, and also for slide representation in image search.

**Results:**

DenseNet’s deep features could distinguish acquisition sites with 70% accuracy whereas KimiaNet’s deep features could reveal acquisition sites with more than 86% accuracy. These findings suggest that there are acquisition site specific patterns that could be picked up by deep neural networks. It has also been shown that these medically irrelevant patterns can interfere with other applications of deep learning in digital pathology, namely image search.

**Summary:**

This study shows that there are acquisition site specific patterns that can be used to identify tissue acquisition sites without any explicit training. Furthermore, it was observed that a model trained for cancer subtype classification has exploited such medically irrelevant patterns to classify cancer types. Digital scanner configuration and noise, tissue stain variation and artifacts, and source site patient demographics are among factors that likely account for the observed bias. Therefore, researchers should be cautious of such bias when using histopathology datasets for developing and training deep networks.

**Supplementary Information:**

The online version contains supplementary material available at 10.1186/s13000-023-01355-3.

## Background

The Cancer Genome Atlas (TCGA) dataset is one of the largest publicly available digital pathology repositories [1]. The TCGA repository contains histopathology whole slide images (WSIs) of 32 cancer subtypes as well as their associated metadata (e.g., clinical information and reports) [2]. Many research groups have trained and validated deep learning models using TCGA images for various tasks ranging from cancer type classification [3–8], tissue segmentation [9, 10] and somatic mutation prediction [3, 5, 8, 11], to survival estimation [4, 12].

One crucial factor that seems to have been widely ignored is the internal bias that originates from the institutions that contributed WSIs to the TCGA dataset. Slides provided by different hospitals may have unique characteristics (e.g., variation in tissue processing, tissue stain protocols, stain quality, color intensity) and other confounding factors including being scanned by specific whole slide scanning hardware platforms, unique imaging protocols (e.g., image compression), and proprietary image file formats. Furthermore, local demographics may lead to batch bias in any given hospital or clinic. The existence of these hospital-identifying factors incorporated into image datasets with accompanying metadata may cause deep models to learn irrelevant visual clues abrogating the generalization of tissue morphology when tested on external validation datasets, i.e., unseen hospitals/clinics.

Image search is a specific application of deep learning in histopathology, where pre-trained deep neural networks (DNN) are used for feature extraction, i.e., feeding images into a pre-trained model and using the deep feature output of a certain layer for image representation [13]. Some researchers have utilized networks, pre-trained on images of non-medical objects [14, 15] such as the ImageNet dataset [16], while other implemented models fine-tuned or trained on pathology images [17–19]. The underlying idea of image search is that deep features extracted from a query image can be compared to all WSIs in a database in a computationally efficient manner, allowing for diagnostic support by locating and retrieving cases of similar morphology. Unfortunately, such an approach may be subject to bias if the feature extractor is trained on specific institutional datasets and potential “hidden” biases are not accounted for. Logically, this bias affects any other operation such as segmentation, prediction, and classification.

Howard et al. showed that the distribution of clinical information in TCGA data such as survival and gene expression patterns significantly differ among samples provided by different institutions [20]. The authors report that a deep learning model utilizing image search could be trained employing histopathology images of six cancer types to accurately discriminate submitting institutions. Additionally, the authors found that it is not possible to effectively obfuscate source sites through common stain normalization techniques. These two findings suggest that some models that claim to predict prognosis or mutation states are perhaps not doing anything more than source site detection. In other words, “biased models” are likely identifying the institution that submitted the sample rather than actually doing what they claim to do. Indeed, DeGrave et al. observed that machine learning models trained on radiographic images have a tendency to learn medically irrelevant shortcuts, usually attributable to biases in data acquisition, instead of the actual underlying pathology [21].

In this report, we used deep features extracted by a deep neural network trained on TCGA WSIs to evaluate biased learning that could lead to distinguishing the source institution that submitted digital slides. This network was trained as a classifier to serve as a domain specific feature extractor for image search in histopathology images.

We address the following questions in this paper:

1. How difficult is it to detect tissue source sites? Is it necessary to explicitly train a deep neural network, or can this be done without training? In other words, does a network pre-trained on non-medical images pick up covert hospital-identifying patterns without any further weight optimization?
2. Does a network, trained for a task that is not equally distributed among contributing medical institutions, learn to distinguish these contributing medical institutions? If true, this suggests that deep networks may use irrelevant clues in the data for decision-making which would erode generalization to images from unseen hospitals.
3. Are tissue source site-identifying patterns detectable in all TCGA projects, rather than just within certain cancer types? If they persist in overall data regardless of cancer type, those patterns are most likely histopathologically irrelevant and unrelated to morphologic similarities between samples provided by each institution.

The lack of generalization is related to the site prediction problem that we investigate. The literature offers a multitude of methods to increase generalization, among others regularization, augmentation, and normalization [22]. These approaches, however, do not provide any insight into the site prediction phenomenon that we investigate in this work.

## Methods

**Fig. 1:**
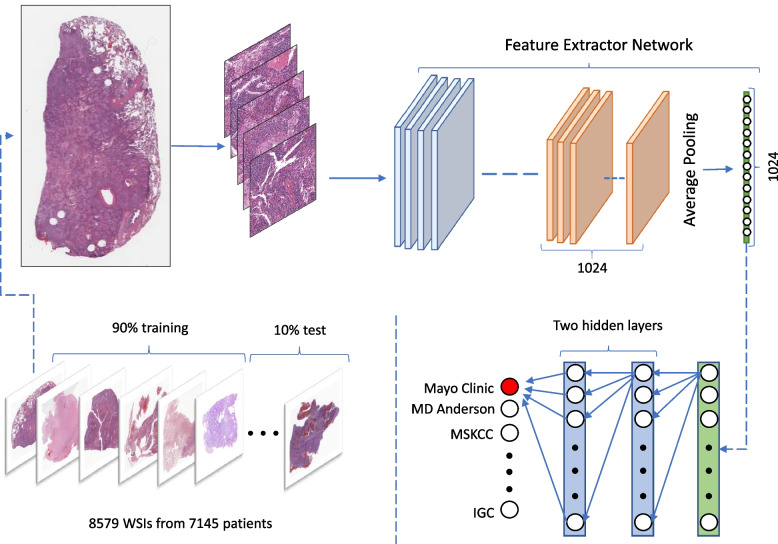
The overview of the training process. WSIs were divided into test and training sets. In our study, 619 patients had more than one WSIs, which were excluded from the test set and only used for training. Tissue patch samples of size 1000 × 1000 pixels derived from WSIs were fed into deep networks (KimiaNet and DenseNet) for feature extraction. The output of the last pooling layer was used as deep features. The extracted deep features were later used to train a smaller network with two hidden layers to identify tissue sources

Acquisition site detection was based on deep features extracted from tissue patches at 20× magnification, using DenseNet121 [23] and KimiaNet [19]. It is important to note that neither of these DNNs had been trained for acquisition site detection. DenseNet was trained on the ImageNet [16] dataset, and KimiaNet was trained for cancer subtype classification of TCGA images. The extracted deep features later were used to train a simple neural network, as a general-purpose optimizer to detect acquisition sites. This process is depicted in Fig. 1.

### Feature extraction

Tissue patches were sampled from each WSI, as per Yottixel paradigm, described by Kalra et al. [15] WSIs were clustered into 9 clusters at 5× magnification based on RGB histograms. Next, tissue patches of size 1000 × 1000 pixels at 20× magnification were selected proportionally to the size of each cluster. Tissue patches with low cellularity were discarded to increase the ratio of tissue patches extracted from malignant regions. More details can be found in the paper published by Riasatian et al. [19] On average, approximately 55 tissue patches were sampled from each WSI.

KimiaNet was selected because the information about its training and test data is publicly available. KimiaNet and DenseNet121 have the same topology, but the former was trained for cancer type classification on TCGA data. Since both our networks have the same structure, deep features were extracted from the same location, the outputs of the last pooling layer of KimiaNet and DenseNet121. Tissue patches were fed to these two DNNs to extract deep feature vectors of size 1024 for each patch. These deep features were separately used for tissue patch representation, and acquisition site classification.

### TSS codes and acquisition sites

All TCGA samples have a unique code starting with “TCGA”, followed by a two-character code called tissue source site (TSS), which is unique to the institution and cancer type. For example, TSS code 02 is for Glioblastoma (GBM) samples provided by MD Anderson. Descriptions of TSS codes are available at this URL address, https://gdc.cancer.gov/resources-tcga-users/tcga-code-tables/tissue-source-site-codes, last accessed on 13/08/2021.

**Fig. 2:**
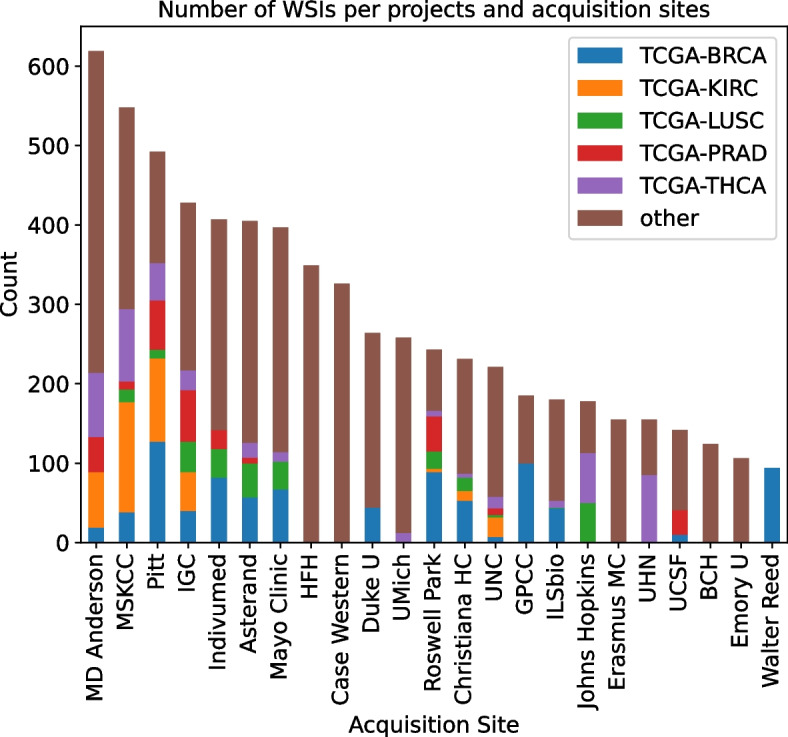
Distribution of cancer types among samples contributed by each group A institution, showing top 5 most frequent cancer types (In TCGA dataset), the rest is labeled as “other”. One could see that the distribution significantly varies among institutions, which can be a source of bias for a model trained for cancer subtype classification. MSKCC: Memorial Sloan Kettering Cancer Center, Pitt: University of Pittsburgh, IGC: International Genomic Consortium, HFH: Henry Ford Hospital, UMich: University of Michigan, UNC: University of North Carolina, GPCC: Greater Poland Cancer Center, UHN: University Health Network, UCSF: University of California San Francisco, BCH: Barretos Cancer Hospital, Duke U: Duke University, Emory U: Emory University, Christiana HC: Christiana Healthcare. KIRC: Kidney Renal Carcinoma, PRAD: Prostate Adenocarcinoma, LUSC: Lung Squamous Cell Carcinoma, BRCA: Breast Carcinoma, THCA: Thyroid Carcinoma

For all diagnostic paraffin-embedded hematoxylin and eosin (H&E) slides, TSS codes were obtained to determine the source institutions. In some cases, naming was inconsistent, therefore we replaced some names. For instance, Memorial Sloan Kettering sometimes was called MSKCC; we replaced MSKCC instances with Memorial Sloan Kettering. We acknowledge that there might still be some inconsistency in institutional naming despite our efforts. This process resulted in 8,579 WSIs from 141 acquisition sites. As shown in Fig. 2, these institutions did not contribute an equal number of WSIs to each TCGA project (cancer type). Therefore, distribution of cases among institutions was imbalanced; 57 institutions provided samples of only one specific cancer type. The real number might be slightly lower than 57 as the tissue source site names were sometimes inconsistent, and we might have mistakenly considered two source sites to be different while they are actually the same. For example, “Emory University” and “Emory University - Winship Cancer Institute” were deemed to be separate institutions.

### Acquisition site classification

It was found that only 24 institutions contributed for more than 1% of the slides, and more than 74% of all slides are from these 24 institutions. In order to prevent institutions with a high number of samples from skewing the results, we divided institutions into two groups. Group A is comprised of acquisition sites with more than 1% of the slides, and group B contains those with fewer slides. Deep feature vectors were subsequently used as the input of a small artificial neural network with two fully connected hidden layers of size 500 and 200 neurons, with rectified linear unit (ReLU) activation function. The models were trained for 5 epochs with a batch size 60, Adam optimizer, and sparse categorical cross-entropy as the loss function. Since the number of acquisition sites was different among each group, the size of each model’s last layer was adjusted with respect to the number of institutions in each group. WSIs were divided into train and test datasets with approximately a 90 to 10 ratio. WSIs of patients that had more than one WSI were only used for training to prevent any confounding interference that might lead to overestimation of classification accuracy. The process of train-test split and training was repeated 30 times to establish a more reliable estimation of classification performance.

### Image search

To undertake the search, we need to calculate the distance between pairs of images represented through their deep features. However, since each WSI is made of several tissue patches, a patch-wise method is required to calculate the distance between the feature vectors of a WSI; a “median-of-min” approach is conducted for this purpose [15]. To calculate the distance between a query image and another WSI, the minimum distance between each patch of the query image and all patches of other WSI is computed first. The median value of all minimum distances was taken as the final distance between the query and compared WSIs. Thus, the most similar images with minimum distance are accordingly identified. However, we also obtained the results of the image search by using the mean of features of all patches within a WSI to compute the distance between WSIs directly. This approach also led to similar results. Having the number of acquisition sites and the number of samples from each institute, an Expected Value (EV) of k can be calculated to show how much we expect that k most similar images will be selected from the same site to which the query image belongs. For this purpose, each sample from the site ${H}_{i}$ is searched among all samples to find the 5 most similar images (i.e., through minimum distance calculations). Then among the search results, we count the number of samples which are selected from ${H}_{i}$.

We acknowledge that due to the absence of pixel-level and regional labels of gigapixel images of large datasets like TCGA, the weak/soft labels are utilized and WSIs are associated with a primary diagnosis for the entire image, which may also include non-cancerous healthy tissue. However, our patch selection pipeline only samples from high-cellular regions which are more likely to be cancerous. Additionally, one of the inclusion criteria of TCGA dataset requires samples to be composed of at least 80% tumor nuclei. (https://www.ncbi.nlm.nih.gov/projects/gap/cgi-bin/study.cgi?study_id=phs000178.v1.p1, last accessed on 14/04/2023). Therefore, our sampled patches are more likely to be dominantly composed of cancerous tissue patches. Ideally, the tissue patches should have only been sampled from cancerous regions to limit confounding factors. However, it requires pixel-level manual annotations which have not been available to us.

## Results

The main purpose of this study is to check if DNNs trained on the TCGA images for a medically relevant task (e.g., cancer subtype classification) learns to detect medically irrelevant patterns. If they do, it implies that that DNN exploits such irrelevant pattern to perform the medically relevant task. In other words, that DNN can be considered biased. In this study we tested acquisition site detection, as the medically irrelevant task that could be helpful to classify cancer subtype classification, the medically relevant task.

### Comparison of KimiaNet and DenseNet121

KimiaNet is a DNN that borrowed the DenseNet-121 topology and was trained on TCGA images to classify 30 cancer subtypes. Whereas DenseNet121 was trained to classify non-medical objects. These two DNNs were used to extract deep features from tissue images. A small artificial neural network (2 hidden layers) was trained on deep features for acquisition site detection. DenseNet121 was evaluated to establish a baseline for the acquisition site detection capacity among TCGA images. An overview of the process is provided in Fig. 1. This process was repeated 30 times to improve the reliability of our results. A comparison between two DNNs may verify whether KimiaNet learned to distinguish patterns unique to each acquisition site or not.

In order to prevent institutions with a high number of samples from skewing the results, we divided acquisition sites into two groups, group A (contributed more than 1% of WSIs) and group B (with fewer WSI contributions). Acquisition site detection was evaluated separately among samples of these two groups. Group B’s results will not be discussed in detail. For the models trained on DenseNet’s deep features, the average classification accuracy was 70% among group A, and 53% for group B institutions. Although these histopathology images have never been seen by DenseNet, this network was able to pick up clues that are useful for identifying acquisition sites. For instance, it was plausible to almost perfectly identify samples provided by “Indivumed Inc”. This finding suggests that there exist some data acquisition-related patterns that could reveal a sample’s acquisition site. Additionally, these patterns could easily be detected by a deep network that were not specifically utilized for pathology images. So, it is plausible to distinguish acquisition sites without an elaborate training process.

For the models trained on KimiaNet’s features, detailed results are shown in Supplementary Table 1. The average accuracy was 86% for group A institutions; and 75% for group B institutions. These results show a 16% and 22% increase in accuracy in group A and B compared to their counterparts trained with DenseNet’s deep features. This finding substantiates the hypothesis that KimiaNet may have inadvertently learned to distinguish tissue source sites that could negatively affect the learning capability of an algorithm. Table 1 shows the average F1-score for the group A among models trained on KimiaNet and DenseNet deep features.

**Table 1:** Comparison of average source site classification performance between KimiaNet’s and DenseNet’s deep features. It suggests that KimiaNet’s deep features contain information pertinent to the tissue source institution

Tissue Source Site Institution	f1-score KimiaNet	f1-score DenseNet	Difference	Number of projects
Indivumed Inc	0.99	0.98	0.01	8
Memorial Sloan Kettering Cancer Center	0.93	0.82	0.11	18
Asterand Bioscience	0.91	0.83	0.08	17
University Health Network	0.91	0.62	0.29	7
Mayo Clinic	0.91	0.70	0.21	9
Barretos Cancer Hospital	0.89	0.61	0.28	7
ILSbio	0.88	0.82	0.06	15
MD Anderson	0.88	0.73	0.15	17
Case Western	0.86	0.72	0.14	2
Johns Hopkins	0.84	0.68	0.16	5
Erasmus MC	0.84	0.67	0.17	4
Roswell Park	0.84	0.68	0.16	18
University of California San Francisco	0.80	0.48	0.32	8
International Genomics Consortium	0.79	0.61	0.18	23
University of Pittsburgh	0.79	0.60	0.19	19
Cureline	0.78	0.56	0.22	10
University of North Carolina	0.75	0.48	0.27	24
Greater Poland Cancer Center	0.70	0.51	0.19	5
Duke University	0.69	0.52	0.17	5
Walter Reed	0.67	0.54	0.13	1
Henry Ford Hospital	0.64	0.32	0.32	2
Christiana Healthcare	0.64	0.50	0.14	17
University of Michigan	0.61	0.29	0.32	6
Emory University	0.41	0.24	0.17	5

**Fig. 3:**
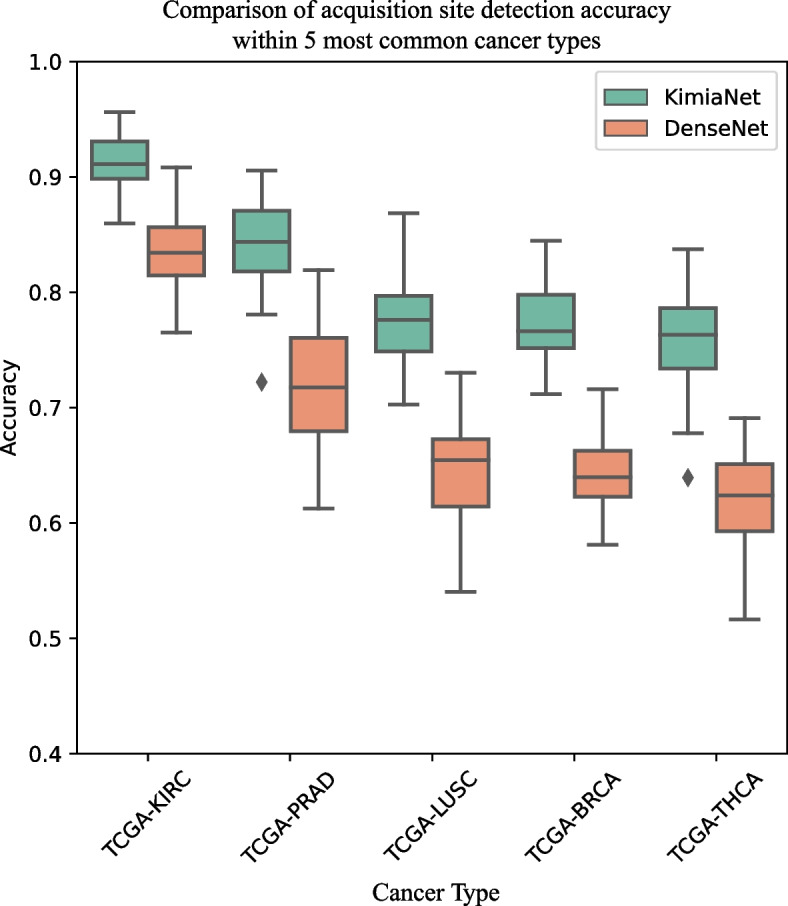
The boxplot of source site accuracies of DenseNet’s and KimiaNet’s deep features over 30 repeats within 5 cancer types with the highest numbers of WSIs. One can see that models trained on KimiaNet’s deep features are consistently more accurate than their counterparts in DenseNet. This finding suggests that KimiaNet’s deep features contain information about source sites of WSIs, although it was originally trained to distinguish cancer types and not source sites. It seems that this additional information, perhaps medically irrelevant, helps the network to classify cancer types due to the TCGA dataset’s internal biases. KIRC: Kidney Renal Carcinoma, PRAD: Prostate Adenocarcinoma, LUSC: Lung Squamous Cell Carcinoma, BRCA: Breast Carcinoma, THCA: Thyroid Carcinoma

An additional hypothesis that may explain the superior performance of KimiaNet versus DenseNet is that KimiaNet can detect source sites because it distinguishes cancer subtypes, and not the other way around. However, it seems unlikely that the distribution of cancer subtypes among tissue source sites is not overly biased. Nevertheless, we tried to train and test for tissue source sites within each of the 30 TCGA projects/cancer types, for detailed results please see Supplementary Table 1. On average, models trained on KimiaNet’s features were 14% more accurate than their counterparts trained on DenseNet’s features. The former set of models were more accurate than the latter within every cancer type, ranging from 1 to 25%. Classification accuracies for 30 repeats within 5 cancer types with the highest number of WSIs are shown in Fig. 3.

**Fig. 4:**
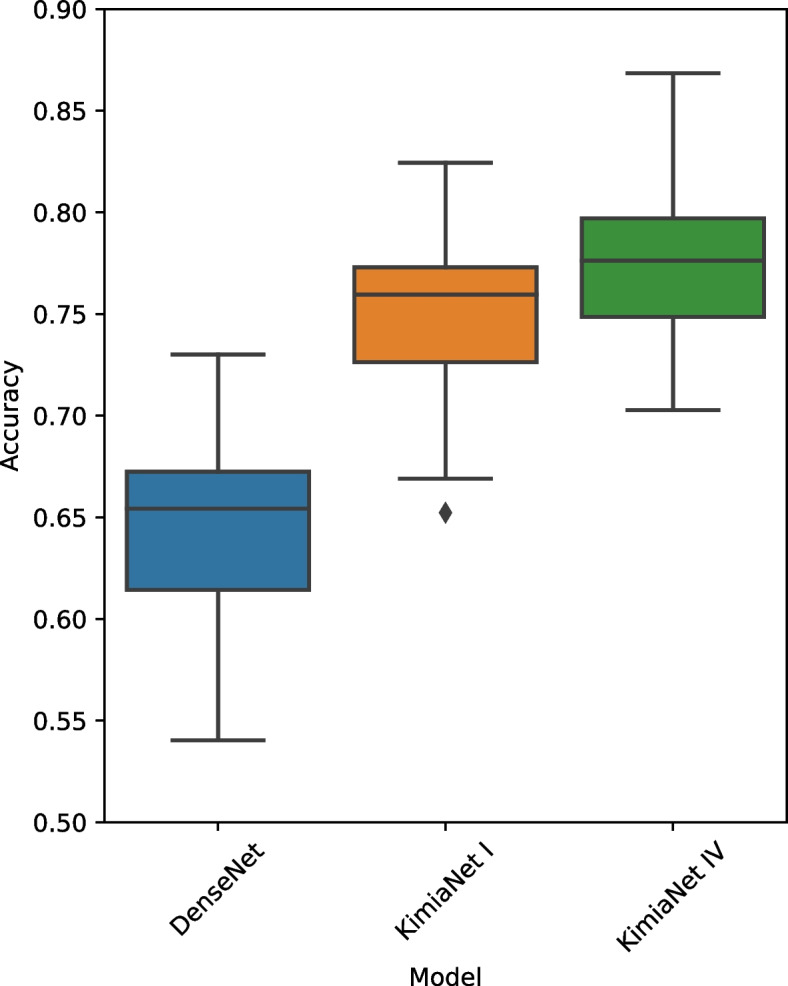
Comparison of acquisition site detection for deep features obtained from DenseNet (trained on nonmedical data), KimiaNet I (finetuned for cancer subtype classification), and KimiaNet IV (fully-trained for cancer subtype classification). The average acquisition site classification accuracy of DenseNet, KimiaNet I, and KimiaNet IV were 0.65, 0.76, and 0.78 respectively. The comparable accuracy of KimiaNet I and IV suggests that site-specific patterns could be easily learned even with fine-tuning

Up until this point, we used deep features from two DNNs, DenseNet trained on nonmedical data, and KimiaNet version IV, where all layers of a DenseNet structure were fine-tuned for cancer subtype classification. Fine-tuning is a popular strategy in machine learning for biomedical image analysis, where only certain layers of a pre-trained network are optimized during the training phase. To compare the ability of fine-tuned networks versus fully trained networks on detecting site-specific patterns, KimiaNet version I was employed, where only the last block of a pre-trained DenseNet was fine-tuned for cancer subtype classification [19]. This experiment was conducted on TCGA-LUSC samples. The average acquisition site classification accuracy of DenseNet, KimiaNet I, and KimiaNet IV were 0.65, 0.76, and 0.78 respectively, please see Fig. 4. This result suggests that site-specific patterns could be easily picked up by the networks even with fine-tuning.

### Impact on image search

Image search, or Content-based image retrieval, is an application of DNNs and deep features in digital pathology. The goal is to search within a database of Images to find images that are the most similar to a query image. KimiaNet is developed to serve as a domain-specific feature extractor for pathology images. Therefore, we also investigated the impact of data acquisition bias on image search. In this series of experiments, we match each query WSI with all WSIs with the same type of primary diagnosis to find the k most similar images. Having the number of acquisition sites and the number of samples from each institute, an Expected Value (EV) can be calculated. For example, if hospital A provided 20% of WSIs, we would expect 1 in 5 search results being from hospital A. Finally, EVs are compared to observed values to discover the possible internal bias toward the institution.

**Fig. 5:**
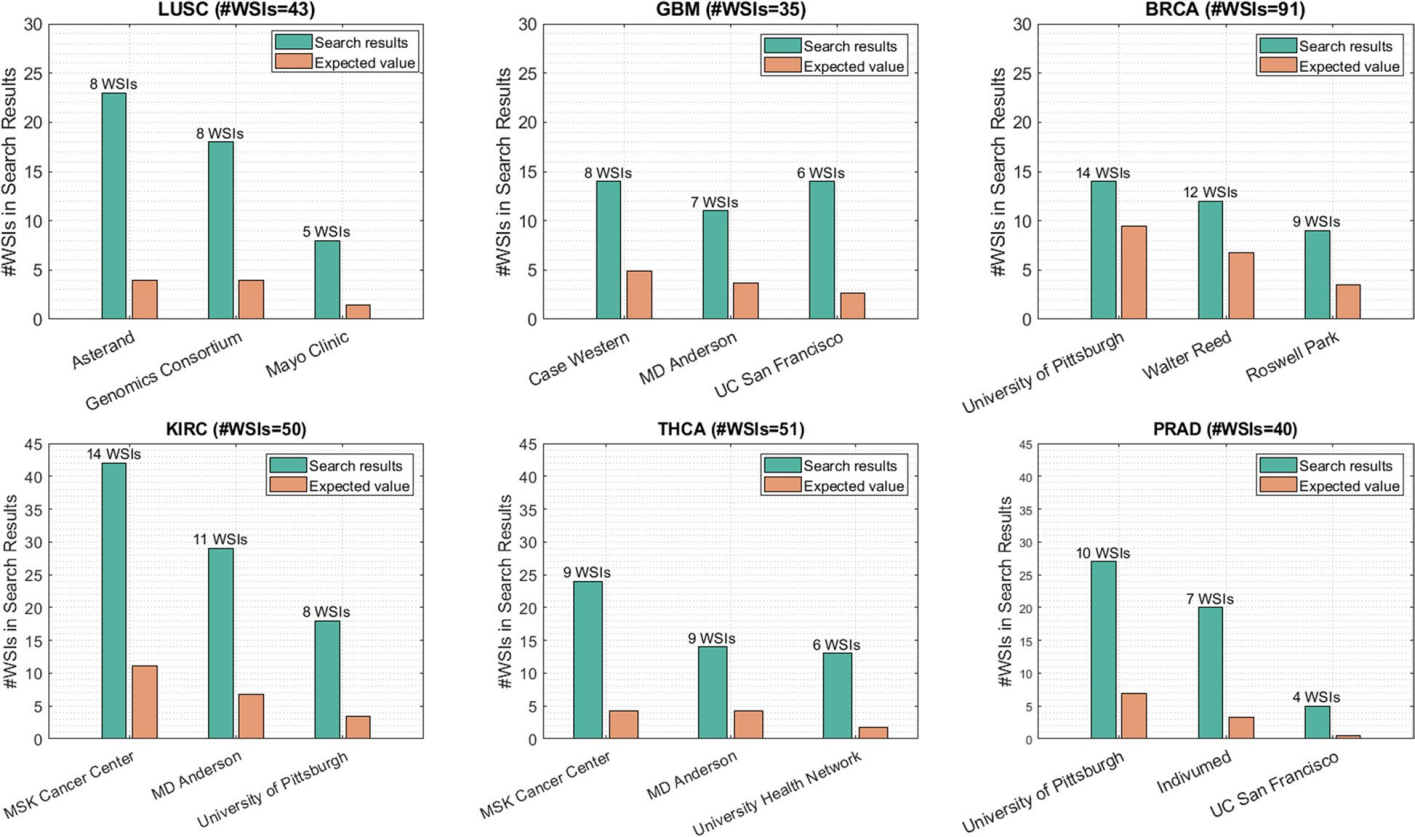
The plots of search results to verify the existence of bias in data. The green bar plots represent the overall number of images in search results from the institute which the query image belongs to. Whereas the orange plots indicate the expected value of this quantity. The number of samples from each institute is presented in the label of the institute. In most of the institutes, the calculated number is more than 4 times higher than the EV

Figure 5 shows the search results on WSIs of six tumor types for three institutions with the highest number of samples. For each institution, the bar plot indicates the number of WSIs found from the corresponding institution while searching for similar images. For instance, on Prostate Adenocarcinoma (PRAD) with 40 WSIs, there are 10 samples from University of Pittsburgh. The five most similar images from those WSIs are selected among all PRAD samples. Therefore, 10 × 5 = 50 images will be the total number of images in search results. One would expect around 10 images to be from the same site in search results. However, 43 out of 50 images are from University of Pittsburgh, which is much higher than the corresponding EV. This suggests the existence of bias towards the patterns extracted based on institution distinction rather than histomorphology of tumor types. In other words, the deep network has discovered hidden patterns in images that distinguish institutions. Consequently, the most similar images of a query image will predominantly be the WSIs from the same site the query WSI originates from.

## Discussion

**Fig. 6:**
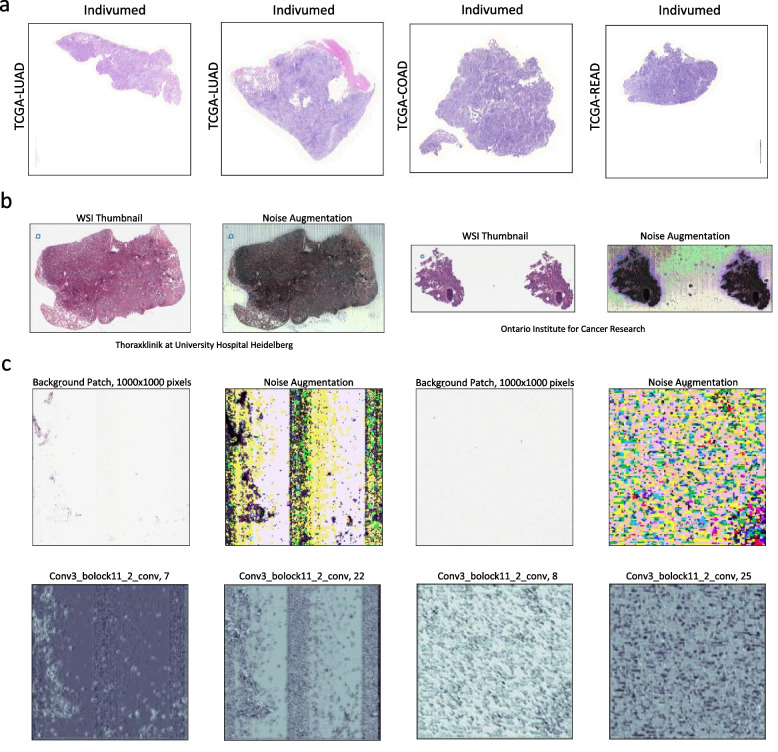
Potential explanations of how deep networks identified tissue source institutions even without explicit training. **a** Tissue patches of WSIs provided by the institution “Indivumed Inc” were accurately identified by using DenseNet’s and KimiaNet’s deep features, perhaps due to their unique stain intensities and color spectrum. One can observe that they have a dominant purplish stain. **b** Digital pathology images are generally noisy. The noise could be amplified by histogram equalization techniques. Although those noisy patterns are more prominent in the background, they most likely exist over the tissue area as well. **c** Convolutional layers could easily pick up noisy patterns existing in a background patch, and over-emphasizing them, as shown by visualizing the output of a convolutional layer of KimiaNet

Participating medical institutions did not contribute equal numbers of cases to TCGA projects (cancer types). Additionally, samples provided by each institution have similar clinical characteristics (e.g., staining techniques, patient demographics, pathology diagnosis). Furthermore, WSIs provided by some institutions may have unique identifiers that could reveal which institution submitted the data. Convolutional neural networks may inadvertently pick up these identifiers or patterns even without any explicit fine-tuning. For instance, DenseNet’s features almost perfectly detected WSIs that originated from “Princess Margaret Hospital” and “Indivumed Inc.” yielding F1-scores of 0.94 and 0.98, respectively. Tissue staining techniques may partly explain this phenomenon as WSIs provided by “Indivumed Inc.” have similar color spectrums regardless of their organ of origin and cancer type, please see Fig. 6a. Such discrepancies could prompt deep models trained on TCGA data to learn tissue source site identifying patterns instead of, or at least in addition to, histopathological morphologic patterns. This was observed when KimiaNet picked up information that is useful for acquisition site detection. For instance, we observed a drastic increase in detection performance for institutions such as “Barretos Cancer Hospital”, “University Health Network”, “Mayo Clinic”. This finding implies that KimiaNet have learned to distinguish source site institutions as a form of undesired (biased) shortcut to classify cancer types. It seems there are complex properties that could be attributed to WSIs provided by each institution, such as slide preparation protocols, hematoxylin and eosin (H&E) stain variation, proprietary image details, and characteristic image noise (see examples in Fig. 6). Noise augmentation has been done by histogram equalization (*equalize_hist* function from python scikit-image [24] library with default parameters), which is a common method for contrast enhancement [25]. Additionally, convolutional layers may easily collect irrelevant noisy patterns of input images, see Fig. 6c. Based on our results, it seems that such patterns could reach deep into the network up until the last layers that we use for image representation.

Although KimiaNet seems to have learned some non-morphologic tissue source site patterns, this is dissimilar to the classic “wolf-versus-husky” problem [26], where a deep network learns to distinguish wolves by just detecting the snow in the background of the image. KimiaNet still needs to learn histologic patterns to distinguish various cancer types provided by a certain institution. For example, it seems KimiaNet can accurately identify WSIs of “Memorial Sloan Kettering Cancer Center”, which contributed samples for 18 various cancer types. Therefore, the knowledge about the source institution does not reveal the diagnosis but it helps to exclude some cancer types. If we assume that such loops exist in the decision-making process of deep networks, they may cause problems in their deployment for clinical utility. If a model mistakenly or correctly attributes a sample to a certain hospital, the reliability of the network’s output will drop. This is not something easily preventable, as convolutional layers easily pick up image noise due to the usage of large number of filters they use. As well, staining techniques may be ineffective in medical institution obfuscation. These issues do not seem to be pertinent to certain topologies, say DenseNet as in our case. Other architectures have shown the same susceptibilities [20].

The most apparent problem when dealing with deep networks in medical image analysis is an apparent overestimation of their performance when trained on TCGA WSIs and perhaps other large WSI repositories. This issue usually manifests itself in a significant performance drop revealed by external validation [27]. Hence, it is advisable to divide training and test data based on the tissue source sites when using TCGA data in order to prevent the model from seeing samples from some institutions and therefore creating a virtual external validation set within TCGA. Although such suggestions may help researchers better estimate their model’s performance without going through the trouble of finding external validation data, it does not address the main issue: deep models may find detours toward easily detectable irrelevant patterns to achieve their goals. Even after excluding some medical institutions from the training data, model learning was impacted by existing biases.

In order to investigate how we can avoid learning site-specific patterns, our research group excluded the participation of images from the same institution during the learning. However, the nature of existing classification-based loss functions does not facilitate the implementation of this idea. Hence, we have worked on a new “ranking loss” function to train the model based on the similarity among images. To this end, we trained an EfficientNet [28] to extract the features for TCGA. Even though with this topology and regular loss function, the bias originating from the institutions is observed, the designed loss function could alleviate the impact of the bias on external validation. By excluding the images of the input hospital from the matched outputs, i.e., sequestering the input domain, the institutional bias can be reduced. The experiments are conducted on the images of two organs, namely brain and lung. The dropping accuracy of institution classification from 73 to 41% for the lung dataset and from 68 to 43% for brain dataset in the proposed model shows that the ranking loss function reduces bias in the trained model to learn the source site institution. In essence, instead of learning source site institutions during the training, the sequestering with ranking loss model concentrates on salient hospital-agnostic image features [29].

It has also been shown that it is not easy to eliminate tissue source site biases through stain normalization techniques [20]. Therefore, the real challenge is to find ways to prevent models from learning transiently useful but histologically irrelevant information. It has also been shown that the distributions of some clinical parameters such as tumor stage, gene expression, receptor status, and survival prognosis are significantly different across tissue source sites. Our finding that a deep network learned to accurately distinguish tissue source sites even though it had not been trained to do so raises the possibility that some research papers published about predicting clinical information from histopathology images without evaluation on external validation data may have been using irrelevant features. One can argue that it is not enough to evaluate deep models on external validation data; we should devise deceptive tests to estimate the robustness of deep models. To give an analogy, it is useful for a handwritten digit recognition model to gain knowledge of who wrote the letter, as everybody’s handwriting has unique subtleties. By exploiting this information, the model would perhaps oversimplify the problem. In extreme cases, this supposed “cheating” prevents the model from recognizing the intrinsically relevant and sensible patterns. Perhaps what such models are learning is tantamount to creating a flawed lookup table with limited (i.e., non-generalizable) success.

As Howard et al. have shown, site-specific patterns/bias may contribute to biased accuracy for other tasks such as predicting survival, genomic mutations, and tumor stage [20]. Bias in healthcare emerges when an AI algorithm takes into account existing social inequities in such as race, ethnicity, socioeconomic in its decision-making process. Therefore, such biased models could eventually exacerbate inequities in healthcare, if being widely implemented [30]. There are examples of AI algorithms being biased toward race or gender in non-healthcare situations [31–34], such as recidivism prediction algorithm that was significantly biased against African American defendants [35]. Since hospitals are located in areas with various demographic characteristics, identification of hospitals not only could cripple a model’s generalizability, but also could introduce social biases into the model’s decision-making process, which would consequently result in the models becoming biased towards demographic characteristics with far-reaching detrimental social consequences.

## Conclusion

In summary, this study showed that TCGA images have unique identifiers that could be used to recognize their respective tissue source hospitals. Convolutional neural networks seem to pick up these identifiers without the need to be explicitly trained to do so. Additionally, a DNN trained for cancer subtype classification has mastered tissue source site identification during its learning phase, and perhaps uses this additional (but irrelevant) information for cancer subtype classification. This phenomenon can negatively affect WSI representation for image classification and search, where categorized images were seen to be disproportionately coming from the same hospital to that of the query image.

## Supplementary Information

**Additional file 1: Supplementary Table 1.** The average performance of source site institution classification over 30 repeats using KimiaNet’s deep features. **Supplementary Table 2.** Comparison between KimiaNet’s and DenseNet’s deep features for classifying tissue source sites within each TCGA project, i.e., cancer type.

## Data Availability

Codes for deep feature extraction and acquisition site detection are available at the following address, https://github.com/TahDeh/TCGA_Acquisition_site_project. Deep features extracted from WSIs used in this study are available from the corresponding author on reasonable request.

## Abbreviations

TCGA: The Cancer Genome Atlas
DNN: Deep Neural Network
WSI: Whole Slide Image
TSS: Tissue Source Site
ReLU: Rectified Linear Unit
EV: Expected Value
KIRC: Kidney Renal Carcinoma
PRAD: Prostate Adenocarcinoma
LUSC: Lung Squamous Cell Carcinoma
BRCA: Breast Carcinoma
THCA: Thyroid Carcinoma
H&E: Hematoxylin and Eosin
