# Supplementary material for: Biased data, biased AI: deep networks predict the acquisition site of TCGA images
Source: Diagn Pathol. 2023 May 17;18:67. doi: 10.1186/s13000-023-01355-3 (PMC10189924; doi:10.1186/s13000-023-01355-3)
Supplement: Supplementary file 1 — Additional file 1: Supplementary Table 1. The average performance of source site institution classification over 30 repeats using KimiaNet’s deep features. Supplementary Table 2. Comparison between KimiaNet’s and DenseNet’s deep features for classifying tissue source sites within each TCGA project, i.e., cancer type. [file 13000_2023_1355_MOESM1_ESM.docx]

**Supplementary Table 1.** The average performance of source site institution classification over 30 repeats using KimiaNet’s deep features.

| Tissue Source Site Institution | Precision | Recall | f1-score | Number of patches | Number of WSIs | Number of projects |
| --- | --- | --- | --- | --- | --- | --- |
| Indivumed Inc | 0.98 | 0.99 | 0.99 | 2918.87 | 61.17 | 8 |
| Memorial Sloan Kettering Cancer Center | 0.93 | 0.92 | 0.93 | 7336.37 | 100.40 | 18 |
| Asterand Bioscience | 0.89 | 0.93 | 0.91 | 5247.90 | 73.73 | 17 |
| University Health Network | 0.91 | 0.91 | 0.91 | 961.17 | 24.83 | 7 |
| Mayo Clinic | 0.91 | 0.91 | 0.91 | 4157.37 | 68.00 | 9 |
| Barretos Cancer Hospital | 0.88 | 0.90 | 0.89 | 1113.97 | 24.20 | 7 |
| ILSbio | 0.9 | 0.86 | 0.88 | 1427.27 | 33.13 | 15 |
| MD Anderson | 0.88 | 0.88 | 0.88 | 5388.80 | 93.10 | 17 |
| Case Western | 0.85 | 0.88 | 0.86 | 890.73 | 22.27 | 2 |
| Johns Hopkins | 0.81 | 0.88 | 0.84 | 1217.50 | 17.33 | 5 |
| Erasmus MC | 0.88 | 0.83 | 0.84 | 932.37 | 13.07 | 4 |
| Roswell Park | 0.83 | 0.85 | 0.84 | 3321.27 | 45.80 | 18 |
| University of California San Francisco | 0.81 | 0.80 | 0.80 | 1264.27 | 24.33 | 8 |
| International Genomics Consortium | 0.79 | 0.79 | 0.79 | 2078.00 | 83.00 | 23 |
| University of Pittsburgh | 0.80 | 0.80 | 0.79 | 6202.27 | 96.80 | 19 |
| Cureline | 0.81 | 0.76 | 0.78 | 741.33 | 17.87 | 10 |
| University of North Carolina | 0.80 | 0.71 | 0.75 | 1512.57 | 38.63 | 24 |
| Greater Poland Cancer Center | 0.63 | 0.80 | 0.70 | 543.4 | 15.40 | 5 |
| Duke University | 0.71 | 0.70 | 0.69 | 581.57 | 8.63 | 5 |
| Walter Reed | 0.76 | 0.62 | 0.67 | 725.57 | 19.27 | 1 |
| Henry Ford Hospital | 0.59 | 0.74 | 0.64 | 135.73 | 20.50 | 2 |
| Christiana Healthcare | 0.60 | 0.70 | 0.64 | 601.23 | 34.73 | 17 |
| University of Michigan | 0.64 | 0.60 | 0.61 | 1124.37 | 13.77 | 6 |
| Emory University | 0.42 | 0.49 | 0.41 | 70.05 | 1.03 | 5 |

**Supplementary Table 2.** Comparison between KimiaNet’s and DenseNet’s deep features for classifying tissue source sites within each TCGA project, i.e., cancer type

| project_id/cancer type | Accuracy KimiaNet | Accuracy DenseNet | Difference | Number of Contributing Medical Centers |
| --- | --- | --- | --- | --- |
| TCGA-ACC | 0.77 | 0.76 | 0.01 | 5 |
| TCGA-BLCA | 0.68 | 0.50 | 0.18 | 33 |
| TCGA-BRCA | 0.77 | 0.64 | 0.13 | 37 |
| TCGA-CESC | 0.70 | 0.45 | 0.25 | 28 |
| TCGA-CHOL | 0.83 | 0.61 | 0.22 | 7 |
| TCGA-COAD | 0.77 | 0.64 | 0.13 | 22 |
| TCGA-ESCA | 0.83 | 0.69 | 0.14 | 18 |
| TCGA-GBM | 0.82 | 0.62 | 0.20 | 14 |
| TCGA-HNSC | 0.79 | 0.66 | 0.13 | 21 |
| TCGA-KICH | 0.97 | 0.73 | 0.24 | 6 |
| TCGA-KIRC | 0.91 | 0.83 | 0.08 | 17 |
| TCGA-KIRP | 0.67 | 0.48 | 0.19 | 31 |
| TCGA-LGG | 0.73 | 0.62 | 0.11 | 22 |
| TCGA-LIHC | 0.66 | 0.54 | 0.12 | 33 |
| TCGA-LUAD | 0.73 | 0.59 | 0.14 | 31 |
| TCGA-LUSC | 0.78 | 0.65 | 0.13 | 33 |
| TCGA-MESO | 0.53 | 0.32 | 0.21 | 11 |
| TCGA-OV | 0.95 | 0.87 | 0.08 | 8 |
| TCGA-PAAD | 0.73 | 0.56 | 0.17 | 19 |
| TCGA-PCPG | 0.68 | 0.49 | 0.19 | 14 |
| TCGA-PRAD | 0.84 | 0.72 | 0.13 | 27 |
| TCGA-READ | 0.82 | 0.76 | 0.06 | 11 |
| TCGA-SARC | 0.78 | 0.62 | 0.15 | 22 |
| TCGA-SKCM | 0.65 | 0.53 | 0.12 | 21 |
| TCGA-STAD | 0.90 | 0.86 | 0.04 | 18 |
| TCGA-TGCT | 0.66 | 0.51 | 0.15 | 15 |
| TCGA-THCA | 0.76 | 0.62 | 0.14 | 21 |
| TCGA-THYM | 0.91 | 0.85 | 0.06 | 6 |
| TCGA-UCS | 0.66 | 0.53 | 0.13 | 8 |
| TCGA-UVM | 0.73 | 0.72 | 0.02 | 5 |
